# Network Analysis of RAD51 Proteins in Metazoa and the Evolutionary Relationships With Their Archaeal Homologs

**DOI:** 10.3389/fgene.2018.00383

**Published:** 2018-09-26

**Authors:** Shan Jiang, Ting Lin, Qingji Xie, Lijing Wang

**Affiliations:** Affiliated Union Hospital, Fujian Medical University, Fuzhou, China

**Keywords:** RAD51, Metazoa, archaea, evolution, network

## Abstract

The RAD51 (DNA repair protein RAD51) recombinases are essential for homologous recombination, DNA repair, and genome stability. Overexpression of RAD51 proteins has been observed in many cancer cells, such as thyroid carcinoma, breast cancer, pancreatic cancer, and others. In Metazoa, there are multiple members of RAD51 (RAD51, RAD51B, RAD51C, RAD51D, DMC1) (DNA meiotic recombinase 1), XRCC2 (X-ray repair cross-complementing 2), and XRCC3. In this study, we used a protein sequence similarity network (SSN) to analyze the evolutionary relationship within this protein family. The SSN based on the RAD51 proteins from Metazoa indicated that there are several proteins that have yet to be functionally defined. The SSN based on the distribution of the proteins supports the hypothesis that horizontal gene transfer plays an important role in the evolution of RAD51 proteins. Multiple sequence alignments with structural information revealed that the amino acid residues for ATP and Mg^2+^ are highly conserved. The seven RAD51 proteins in humans are under different selective pressure: RAD51 and DMC1 are under stringent negative selection, while other proteins are subject to relatively relaxed negative selection. Furthermore, the expression levels of the seven genes in different tissues showed that the genes in the same cluster in the phylogenetic tree showed similar expression profiles. Finally, the SSN based on the RAD51 proteins from both eukaryotes and prokaryotes suggested that the eukaryotic RAD51 recombinases share a common ancestor with the archaeal homologs, but XRCC2 may have a different origin. These findings expand the understanding of the evolution and diversity of RAD51 recombinases in Metazoa.

## Introduction

Homologous recombination (HR), the exchange of DNA strands of similar or identical nucleotide sequence, is essential to cell division in eukaryotes, including protists, fungi, plants, and animals. HR is also an essential process in the repair of many structurally diverse DNA lesions, such as collapsed replication forks, interstrand crosslinks, and double-strand breaks (Dudás and Chovanec, 2004; Holthausen et al., 2010). The key step of HR is an ATP-dependent DNA-strand exchange that is mediated by a family of conserved recombinases, such as RAD51 and RecA, that mediate a homology search and DNA strand exchange through the formation of a dynamic nucleoprotein filament (Li and Heyer, 2008; Yeeles and Dillingham, 2010).

The RAD51 protein plays a central role in the stability of the genome and the normal cell cycle. The expression of RAD51 increases in S and G2 phases of the cell cycle, which correlates with the predominant activity of HR observed in these stages of the cell cycle (Shrivastav et al., 2008). Strikingly, recent studies have indicated that the RAD51 protein is overexpressed in various types of tumors, including those in breast cancer, non-small cell lung cancer, cervical cancer, pancreatic cancer, ovarian cancers, melanoma, and glioblastoma (Richardson, 2005; Nagathihalli and Nagaraju, 2011). RAD51 overexpression may lead to improper hyperrecombination, which might drive regular cells toward neoplastic transformation or further contribute to cancer progression and metastasis (Hine et al., 2008; Chen et al., 2017). Tumor cells that overexpress RAD51 show increased cell survival, hyperrecombination, resistance to radiation, and various types of drugs that are known to induce DNA double-strand breaks (Nagathihalli and Nagaraju, 2011). Considering the importance of RAD51, it has been considered as a potential surrogate marker for DNA repair capacity in solid malignancies (Gachechiladze et al., 2017).

RAD51 in eukaryotic cells, together with RADA in archaea and RecA in bacteria, belong to a RecA/RAD51 superfamily of conserved recombinases that catalyze ATP-dependent pairing and strand exchange between homologous DNA molecules (Prentiss et al., 2015). Bacteria generally have one *RecA* gene. There are two *recA/RAD51*-like genes, called *RADA* and *RADB*, in several archaeal species. In yeast, four eukaryotic *RAD51*-like genes (*RAD51*, *DMC1*, *RAD55/rhp55*, and *RAD57/rhp57*) exist. Vertebrate animals and plants usually have seven different *RAD51*-like genes: *RAD51*, *RAD51B*, *RAD51C*, *RAD51D*, *DMC1*, *XRCC2*, and *XRCC3*. Additionally, four conserved *RecA*-like genes that have higher levels of sequence similarity with eubacterial *RecA* genes than with the eukaryotic *RAD51*-likes genes can be found in flowering plants, such as rice (*Oryza sativa*) and *Arabidopsis thaliana* (Lin et al., 2006). In this current research, we performed a comprehensive analysis using multiple sequence alignments (MSAs), sequence similarity networks (SSNs), and phylogenetic analyses to gain insights into the evolution and diversity of the RAD51 family in Metazoa.

## Materials and Methods

### Collection of Functional and Putative RAD51 and the Homologs

For a global analysis of the RAD51 protein family in Metazoa, the sequences of these genes were retrieved from the InterPro database^1^ (release 68.0) (Mitchell et al., 2015). Redundant sequences were removed by CD-HIT, with a requirement of 100% identity for the SSN of Metazoan proteins and 90% identity for an SSN of all of the proteins from the InterPro database (IPR013632) (Li and Godzik, 2006). The proteins were then screened for the presence of the RAD51 domain using the Pfam database (Marchler-Bauer et al., 2015). The proteins identified in the database are listed in the **Supplementary Dataset**.

### Construction of SSNs

Sequence similarity networks were constructed using the Enzyme Function Initiative-Enzyme Similarity Tool (EFI-EST) (Gerlt et al., 2015) and visualized using Cytoscape 3.3 (Shannon et al., 2003). The input sequences were obtained from the Interpro database. Each node in the network indicates a protein, and an edge indicates that the two nodes connected by that edge are significantly similar, having an e-value less than the selected cutoff (Jia et al., 2017).

### Multiple Sequence Alignments (MSAs) and Coevolving Protein Residues

Multiple sequence alignments of protein sequences were performed using the MAFFT (version 7) program (Katoh et al., 2017). The unrooted phylogenetic trees were constructed with MEGA7 using maximum likelihood (ML) methods and bootstrapping with 1000 iterations (Kumar et al., 2016). Analysis of coevolving residues was carried out using mutual information (MI) between two positions in the MSA, which reflects the extent to which knowledge of the amino acid at one position can allow prediction of an amino acid at other positions. MI was calculated between pairs of columns in the MSA using the MISTIC web server (Simonetti et al., 2013). Protein structures were retrieved from the PDB database (PDB ID: 5H1B) and visualized by PyMOL^2^.

### Protein Expression

The gene expression data used in this study were retrieved from The Human Protein Atlas (HPA) (Uhlen et al., 2015).

### Estimation of *K*_a_/*K*_s_ Ratio

*K_a_/K_s_* or dN/dS indicates the ratio of non-synonymous to synonymous substitution rate. The ratio was calculated for the RAD51 of both human and mouse using PAL2NAL (Suyama et al., 2006).

## Results

### Diversity and Distribution and of RAD51 Proteins in Metazoa

**FIGURE 1:**
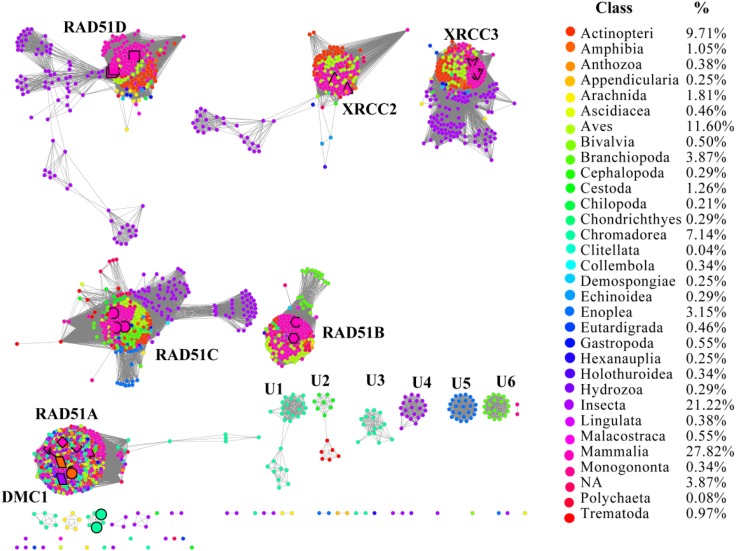
The protein sequence similarity network (SSN) and taxonomic distribution (classes) of RAD51 from Metazoa. The SSN of proteins in the InterPro database (IPR013632) was generated with an e-value threshold of 10^-40^. Each node represents one protein. Edges are shown with BLASTP e-values below the indicated cutoff. A cluster was labeled if there were more than 10 nodes in it. RAD51A (diamond), RAD51B (hexagon), RAD51C (octagon), RAD51D (square), XRCC2 (triangle), XRCC3 (V), and DMC1 (parallelogram) are enlarged. The proteins that are annotated with other names are also enlarged and shown in cycle forms. The clusters without any annotated proteins are labeled sequentially as U1 to U6. Nodes from the same taxonomic groups in the global network have the same color. The colors corresponding to each class and protein percentage in each class are listed at the right.

RAD51 contains an α-helical DNA binding domain in the N-terminus (Pellegrini et al., 2002). We collected 2,378 homologs of RAD51 from the Interpro protein database for Metazoa, which were annotated in the Pfam protein database^3^ (**Supplementary Dataset**). The SSNs for these 2,378 sequences were constructed using the e-value cut-offs of 10^-20^, 10^-30^, and 10^-40^, at which >25, >30, and >40% sequence identity was required to draw an edge between nodes, respectively (**Figure 1** and **Supplementary Figure S1**) (Jia et al., 2017). At an e-value threshold of 10^-20^, almost all the proteins were located in two clusters that were connected by one edge; one cluster contained XRCC2 from humans and its homologs and another cluster contained other human proteins, including RAD51A, RAD51B, RAD51C, RAD51D, DMC1, and XRCC3, which indicated that XRCC2 had low sequence identity with other proteins. As the e-value cut-off was decreased to 10^-30^, human RAD51D was further separated from the cluster, but other proteins from humans (RAD51A, RAD51B, RAD51C, DMC1, and XRCC3) were still grouped together. As the e-value threshold stringency was further decreased to 10^-40^, the seven experimentally characterized proteins could be further separated into six clusters. RAD51A and DMC1 were close to each other, indicating that they share high identity in the protein sequence, and they were duplicated from the same ancestor. Additionally, there were six clusters containing 10 or more members that have been annotated in the literature or in Swiss-Prot, suggesting that the proteins in the six clusters may have new functions that are different from the known seven RAD51 proteins. In conclusion, the SSN analysis indicated that the majority of RAD51 recombinases are known, and their function may be assigned to canonical classes by means of comparison with previously characterized proteins. The results also suggested that RAD51A and DMC1 were duplicated from the same ancestor, but that XRCC2 was far from other proteins in terms of evolution.

To explore the occurrence of RAD51 recombinases in Metazoa, the network was coded by taxonomic classification (**Figure 1**). Members of the RAD51 family were found in 31 classes of the kingdom Metazoa. However, the relative abundance of RAD51 proteins varied widely among the classes. The prevalence of *RAD51* genes was found to be high in *Mammalia*, which accounted for 27.8% of all the *RAD51* genes examined. *Insecta* showed the second highest abundance (21.2%), followed by *Aves* (11.6%), *Actinopteri* (9.7%), and *Chromadorea* (7.1%). The proteins in clusters without any experimentally characterized proteins were mainly from the classes of *Chromadorea*, *Insecta,* and *Branchiopoda*. Notably, the proteins in the same class were always close to each other within one cluster, except for the RAD51A/DMC1 group. The same phenomenon was also observed in the SSN at the phylum level (**Supplementary Figure S1**), which suggests that the evolution of *RAD51* genes in Metazoa has been mostly determined by gene duplication and vertical gene transfer.

### Consensus and Coevolution of Amino Acid Residues in RAD51 Proteins

**FIGURE 2:**
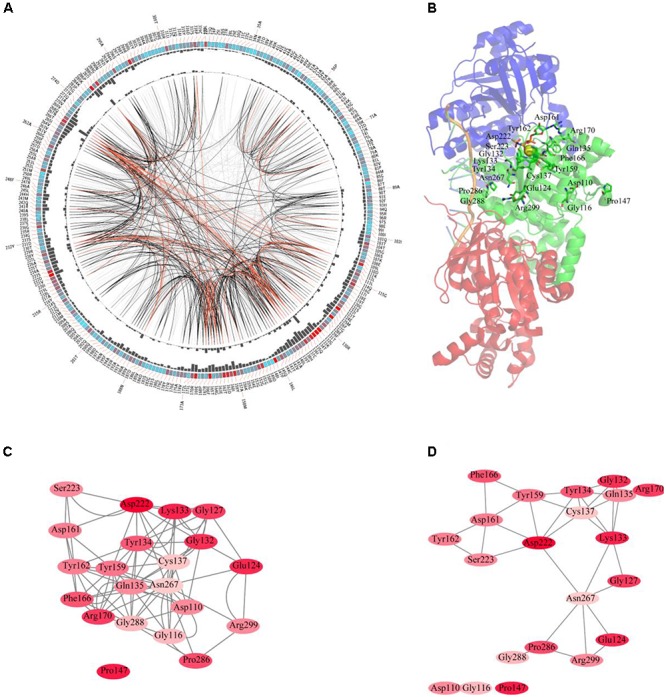
Conserved and coevolved amino acid residues in RAD51 represented by RAD51A as a reference sequence. **(A)** Network analysis of coevolving residues. The circular network shows the connectivity of coevolving residues. The labels in the first circle indicate the alignment position and the amino acid code of RAD51A. The colored square boxes of the second circle indicate the multiple sequence alignment (MSA) position conservation (highly conserved positions are red, whereas less conserved ones are blue). The third and fourth circles show the proximity mutual information (MI) and cumulative MI (cMI) values as histograms, facing inward and outward, respectively. In the center of the circle, the edges that connect pairs of positions represent a significant MI value (>6.5), highlighted with red lines to show a higher MI score (top 5%), black ones for a moderately high MI score (between 70 and 95%), and gray ones for a lower MI score (the remaining 70%) as defined by MISTIC. **(B)** A cartoon diagram of RAD51A (PDB ID: 5H1B) with the top conserved amino acid residues. **(C)** The network of cMI values of amino acid residues with a high conservation value. Nodes represent top conserved amino acid residues (labeled with a position and code) and are colored by conservation from pink (lower higher) to red (higher). The length of the edges is inversely proportional to the MI value (the closest nodes have higher MI). **(D)** The network of distances of amino acid residues with a high conservation value. Nodes represent top conserved amino acid residues (labeled with a position and code) and are colored by conservation from pink (lower higher) to red (higher). Edges are presented as lines binding nodes if they are closer than 5 Å (the closest nodes have a smaller distance).

The sequences of RAD51 proteins were diverse based on the network analysis. To examine if the protein sequences were conserved across evolution, the consensus sites of RAD51 proteins were analyzed using MSAs. The protein sequence of RAD51 from humans, with UniProt ID Q06609, was used as the reference sequence for MSAs, and the conservation of the residues to this sequence are shown in **Figure 2A**. The highly conserved residues were mapped on the structure of human RAD51A (**Figure 2B**) and further analyzed by amino acids coevolution (**Supplementary Figure S2**).

RAD51 and its bacterial counterpart, RecA, share a highly conserved, ∼230 amino acid domain that contains two putative ATP-binding domains, the Walker A (also called A-site for NTP binding) and Walker B (also called A-site for Mg^2+^ chelating) motifs. DNA-binding activity of hRAD51 is attributed to its N-terminal domain, which includes a modified helix–hairpin–helix (HhH) motif (Suzuki et al., 2012). Based on the MSAs, the Walker A and Walker B motifs are highly conserved. Among them, Asp222 was the most conserved residue, which was located in the Walker B motif. Asp222 is responsible for binding Mg^2+^, and mutation of D222 to Alanine greatly impedes the ability to bind ATP (Morrison et al., 1999). In Walker motif B, Asp222 is sequestered in a solvent-inaccessible hydrogen-bonding network that extends to Tyr159, Tyr162, Asp161, and Thr165 via a buried water molecule (Davies and Pellegrini, 2007). Among these residues, Tyr162 and Asp161 are also highly conserved. In the Walker A motif, Lys133, Glu124, and Gly132 were the most conserved residues. The ATPase activity of RAD51 is abrogated by the K133R mutation (Morrison et al., 1999). Although NMR studies show that residues Ala61–Glu69 in the HhH motif of RAD51 may bind dsDNA (Aihara et al., 1999), these residues were not highly conserved based on MSAs. In contrast, Pro147 is also highly conserved, but its function has not been determined yet. Compared with the amino acid residues in the N-terminus required for ATP-binding and hydrolysis, the residues in the C-terminus were less conserved. It has been reported that the C-terminus is responsible for RAD52 binding. This binding can promote homologous pairing between ssDNA and dsDNA and higher homologous pairing activity has been observed in the presence of both HsRAD51 and HsRAD52 than with either HsRAD51 or HsRAD52 alone. Among the amino acids required for binding, Pro289 and Arg299 were highly conserved, and R299Q was significantly defective in binding RAD52 (Kurumizaka et al., 1999). For clearer illustration of this, the top conserved residues were further mapped onto the structure of RAD51. The results revealed that most of the conserved amino acids form a close cluster surrounding NTP and Mg^2+^ (**Figure 2B**).

We further studied the coevolution of amino acid residues in RAD51 using MI (**Figure 2A**). If two residues have a high MI score, then they most likely have been coevolving, meaning that a mutation of one amino acid is linked to a specific compensatory mutation of the other one to maintain a given enzymatic function (Jia et al., 2017). The MI network for the RAD51 proteins revealed that higher MI values (top 10% of MI values) belonged to the highly conserved amino acid positions (**Figure 2A**). A strong correspondence between conserved residues and coevolving residue positions in the substrate and cofactor-binding sites was consistent with previous studies (Yeang and Haussler, 2007; Tse and Verkhivker, 2015). The 20 top-scoring residues formed a connected network, indicating these residues share a significant MI value (**Figure 2C**). The distance network showed that these amino acids were also closed to each other in the structure (**Figure 2D**). These analyses indicated that these amino acids in RAD51 were highly conserved and coevolved during evolution.

### Effects of Mutagenesis on the Function of RAD51A

The effects of the mutagenesis of several conserved amino acids, such as K133R, D222A, and R299Q, on the function of human RAD51A were studied. The three amino acids are located at the Mg^2+^ binding motif, ATP binding motif, and RAD52 binding motif, respectively (**Table 1**). In addition to the three amino acids, other residues that were less conserved also affect the function of RAD51A in humans. For example, K58R with K64R impaired the ubiquitination of the enzyme (Masuda-Ozawa et al., 2013). Both F86A and A89E cause the loss of homooligomerization of RAD51A. Mutagenesis at the C-terminus (S208A:A209E) disrupts the interaction between RAD51A and BRCA2. RAD51 mutagenesis is also associated with several diseases. The natural variants D149N, R150Q, and G151D show low ATP hydrolysis activity. These mutants also alter the physical properties of nucleoprotein filaments. These mutants may also contribute to genome instability in breast tumor cells (Chen et al., 2017). Furthermore, the natural variants T131P and A293T are associated with Fanconi anemia, a hereditary disease featuring hypersensitivity to DNA cross-linker-induced chromosomal instability (Ameziane et al., 2015). These mutagenesis studies indicated that the amino acids in human RAD51A are important for its function, even if they were less conserved based on MIs.

**Table 1:** Effect of mutagenesis on the function of RAD51A from humans.

Feature key	Mutagenesis	Effect on functions	Reference
Mutagenesis	K58R	Impaired ubiquitination; when associated with R-64	Masuda-Ozawa et al., 2013
	K64R	Impaired ubiquitination; when associated with R-58	Masuda-Ozawa et al., 2013
	F86A	Loss of homooligomerization	Pellegrini et al., 2002
	A89E	Loss of homooligomerization	Pellegrini et al., 2002
	K133R	ATPase activity is abrogated	Morrison et al., 1999
	S208A:A209E	Disrupts interaction with BRCA2	Yu et al., 2003
	D222A	Impede the activity to bind ATP	Morrison et al., 1999
	R299Q	Defective in binding RAD52	Kurumizaka et al., 1999
	T309A	Confers hypersensitivity to hydroxyurea	Sorensen et al., 2005
Natural variant	D149N	Mutations are associated with human breast tumors. These mutations show low catalytic efficiency for ATP hydrolysis and also alter the physical properties of RAD51 nucleoprotein filaments.	Chen et al., 2017
	R150Q		
	G151D		
	T131P	The mutations are associated with Fanconi anemia. The mutations impair function in DNA repair	Ameziane et al., 2015; Wang et al., 2015
	A293T		


### Selection Pressure on RAD51 Coding Sequences

The ratio of non-synonymous (*dN*) to synonymous (*dS*) nucleotide substitution with pairwise or multiple combinations of genes can be used to identify the selection pressure on genes. Under neutrality, the ratio is not expected to deviate significantly from 1 (*dN/dS* = 1), whereas notable changes in this ratio may be interpreted as evidence of positive selection (*dN/dS* > 1) or negative selection (*dN/dS* < 1). We generated a pairwise codon alignment between human and *Mus musculus* using PAL2NAL and using the full-length sequences of the seven RAD51 proteins (**Table 2**). We observed that the *dN/dS* values for all these genes was <<1, which indicated that the RAD51 recombinases were under negative selection. However, the values for the seven genes were different: the *dN/dS* values for both RAD51 and DMC1 were <0.05; the *dN/dS* value of RAD51 was only 0.017; yet, the values for other the genes were approximately 0.20. These results indicated that RAD51 and DMC1 were under stringent negative selection while other RAD51 proteins in humans were subject to relaxed negative selection.

**Table 2:** Tabular representation of the dN/dS calculations between human and *Mus musculus* in seven RAD51 proteins.

Protein	*Number of synonymous site (S)*	*Number of non-synonymous site (N)*	*Synonymous substitution rate (dS)*	*Non-synonymous substitution rate (dN)*	*dN/dS*
RAD51	300.7	716.3	0.4112	0.0071	0.0172
RAD51B	308.5	741.5	0.3983	0.0855	0.2147
RAD51C	303.3	794.7	0.3820	0.0694	0.1816
RAD51D	298.8	685.2	0.5452	0.1034	0.1896
XRCC2	230.7	600.3	0.7373	0.1311	0.1778
XRCC3	263.8	774.2	0.9668	0.1704	0.1763
DMC1	263.3	756.7	0.3681	0.0159	0.0432


### Expression of *RAD51* Genes in Humans

**FIGURE 3:**
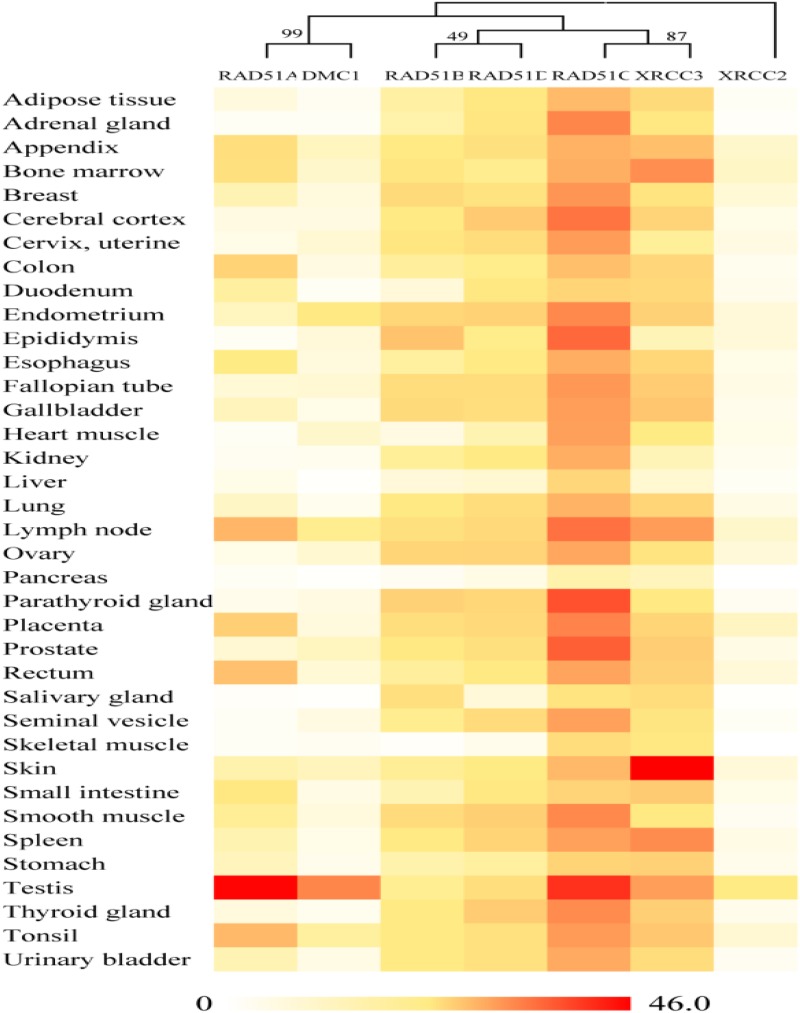
Gene expression patterns of RAD51 in humans using RNA-Seq data from the Human Protein Atlas database (HPA). RNA-seq gene expression profiles for 7 human RAD51 proteins across 37 human tissues. Mean Expression Level indicates the mean, prenormalization mRNA expression level of each RAD51 (in TPM) across all tissues.

On the basis of publicly available microarray data from the human ATLAS database, *RAD51* genes in humans are expressed in various tissues (**Figure 3**). Nevertheless, these genes showed distinct expression profiles, which were related to their evolutionary relation. *RAD51* and *DMC1* ended up in the same cluster. These two genes are highly expressed in the testis. *RAD51* is also expressed in the lymph node, tonsil, and rectum. In the phylogenetic tree, *RAD51B* and *RAD51D* fall into a different cluster. These two genes were widely expressed in different tissues except for in skeletal muscle. Similarly, *RAD51C* and *XRCC3* clustered into a clade together in the phylogenetic tree and were also expressed highly in various tissues. Finally, *XRCC2* was separated from other proteins in the phylogenetic tree and its expression in tissues was also relatively low compared to other *RAD51* genes. These data suggests that the expression of *RAD51* genes is closely related to their evolution.

### Evolutionary Relationship of RAD51 Proteins in Metazoa and Archaea

**FIGURE 4:**
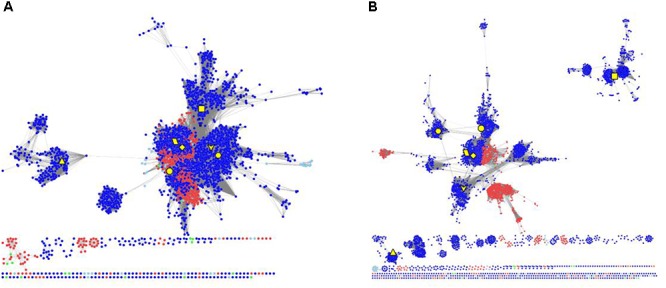
The protein SSN of RAD51 from eukaryotes and prokaryotes. The SSN of proteins was generated with an e-value threshold of 10^-20^ **(A)** and 10^-35^ **(B)**. Each node represents one protein. Edges are shown with BLASTP e-values below the indicated cutoff. RAD51A (diamond), RAD51B (hexagon), RAD51C (octagon), RAD51D (square), XRCC2 (triangle), XRCC3 (V), and DMC1 (parallelogram) from humans are enlarged and showed in yellow. Other proteins from Metazoa are shown in blue, and the proteins in archaea are shown in red. The proteins from bacteria and metagenomes are shown in green and blue, respectively.

To elucidate the evolutionary relationship of RAD51 proteins in Metazoa and other organisms, we performed extensive searches for RAD51-like genes from Interpro databases and performed SSN analyses of the genes identified (**Figure 4** and **Supplementary Figure S2**). The results showed that the seven Metazoan RAD51 proteins, except for XRCC2, were quite similar to the proteins from archaea, but not bacteria, when the e-value was set to 10^-20^ (>30% sequence identity was required to draw an edge between nodes). If the cut-value of protein identity was set to 35% (e-value = 10^-25^), XRCC2 was separated from the clusters, including the proteins from both Metazoa and archaea. RAD51D and its homologs were the second cluster to be separated after the cut-off was increased to 40% (e-value = 10^-30^). At this value, RAD51A and DMC1 were still clustered closely with the homologs from archaea. RAD51B, RAD51C, and XRCC3 were also located in the same cluster, but clustering of these three proteins’ homologs were not as close as clustering between RAD51A/DMC1 and archaeal RAD51. The network analysis suggests that RAD51A/DMC1 in Metazoa are very close to archaeal homologs and that XRCC2 proteins are quite far from other proteins across evolution.

## Discussion

In this study, a large-scale *in silico* analysis of RAD51 in Metazoa was performed. The SSNs revealed that most clusters of RAD51 proteins share more than one protein that has been characterized experimentally. The SSNs also suggested that gene duplication and vertical gene transfer play important roles to driving RAD51 protein evolution. Our results also indicated that residues directly involved in ATP and Mg^2+^ binding were highly conserved and have coevolved. The seven *RAD51* genes in humans are under different evolution pressures, and their expression profiles are correlated with their evolution. Finally, the RAD51 in eukaryotes were much closer to homologs in archaea, but not in bacteria.

The *RAD51/DMC1* genes may have been generated by an ancient gene duplication, as demonstrated by evidence of a common ancestor of all eukaryotes (DiRuggiero et al., 1999). Lin et al. (2006) classified the eukaryotic homologs of RAD51 into RADα (including RAD51 and DMC1) and RADβ (including RAD51B, RAD51C, RAD51D, XRCC2, and XRCC3). Because the RADα and RADβ subfamilies each contain archaeal and eukaryotic members, a gene duplication event to generate RADα and RADβ was suggested to occur before the archaea/eukaryote split. Gene duplication produced the *RAD51* and *DMC1* genes from RADα, whereas the RADβ group generated the *RAD51C*, *XRCC3*, *RAD51B*, *RAD51D*, and *XRCC2* genes successively (Lin et al., 2006). Our network analysis supports the hypothesis that a gene duplication event occurred before the archaea/eukaryote split, because RAD51/DMC1 shared high sequence identity with archaeal homologs, and the archaea generally have two copies of *RAD51* genes (**Figure 1**). However, the networks also showed that RAD51, DMC, RAD51C, XRCC3, RAD51B, and RAD51D were very close, while XRCC2 was quite far from these proteins. The expression profile of *XRCC2* is also different from other genes (**Figure 1**). Therefore, we suggest that XRCC2 may not share a common ancestor with other RAD51 recombinases, and the evolution of XRCC2 will require further examination in future work.

## Author Contributions

SJ and LW designed the experiments, analyzed all the data, and wrote the manuscript. TL and QX analyzed the gene expression patterns. All authors reviewed the manuscript.

## Conflict of Interest Statement

The authors declare that the research was conducted in the absence of any commercial or financial relationships that could be construed as a potential conflict of interest.

## Footnotes

1 http://www.ebi.ac.uk/interpro/entry/IPR013632

2 https://pymol.org/

3 http://pfam.xfam.org/
