# Supplementary material for: Network Analysis of RAD51 Proteins in Metazoa and the Evolutionary Relationships With Their Archaeal Homologs
Source: Front Genet. 2018 Sep 26;9:383. doi: 10.3389/fgene.2018.00383 (PMC6168637; doi:10.3389/fgene.2018.00383)
Supplement: Supplementary file 1 [file Table_1.DOCX]

**Network analysis of RAD51 proteins in Metazoa and the evolutionary relationships with their archaeal homologs**

**Figure S1A**


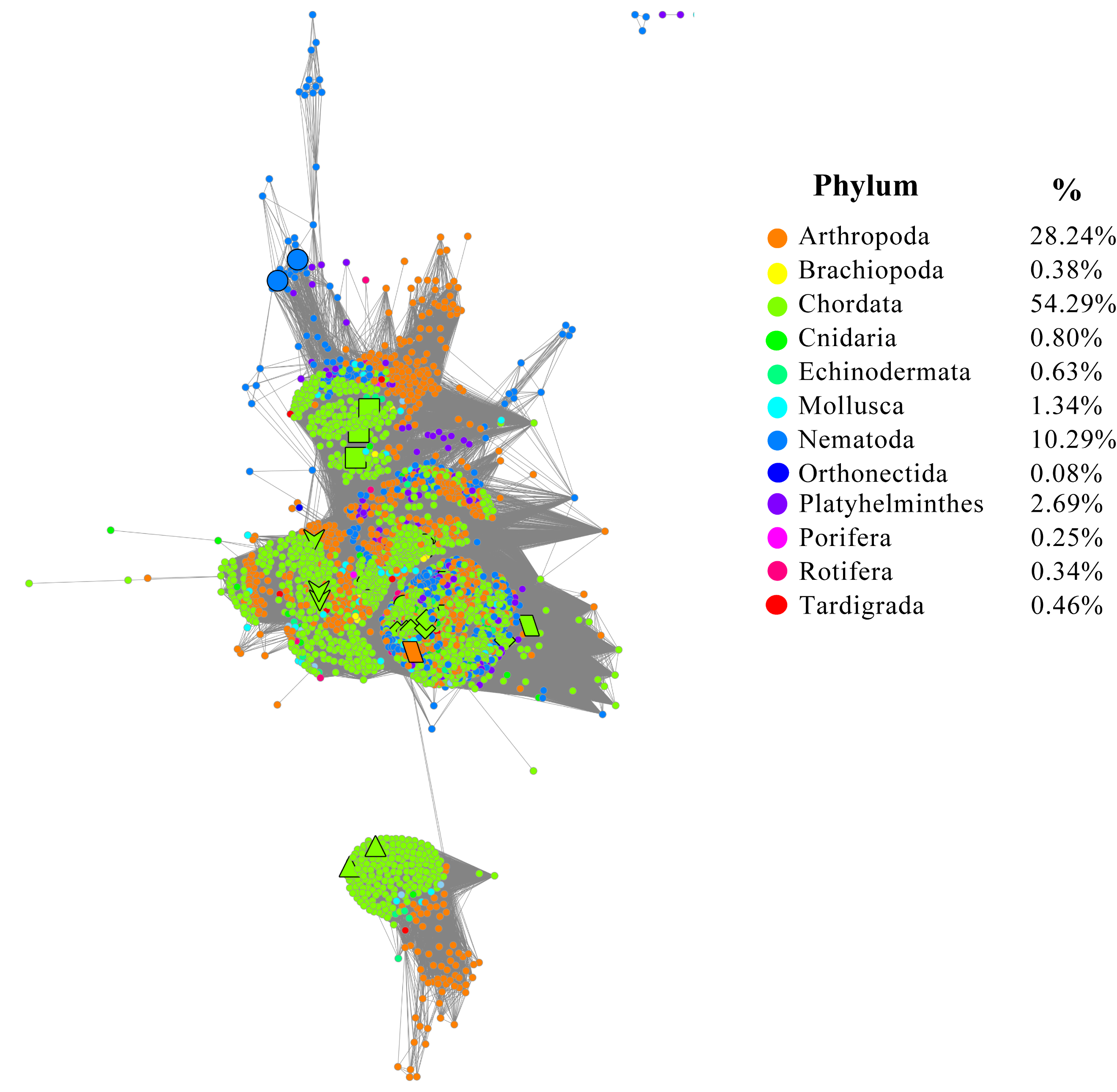


**Figure S1B**


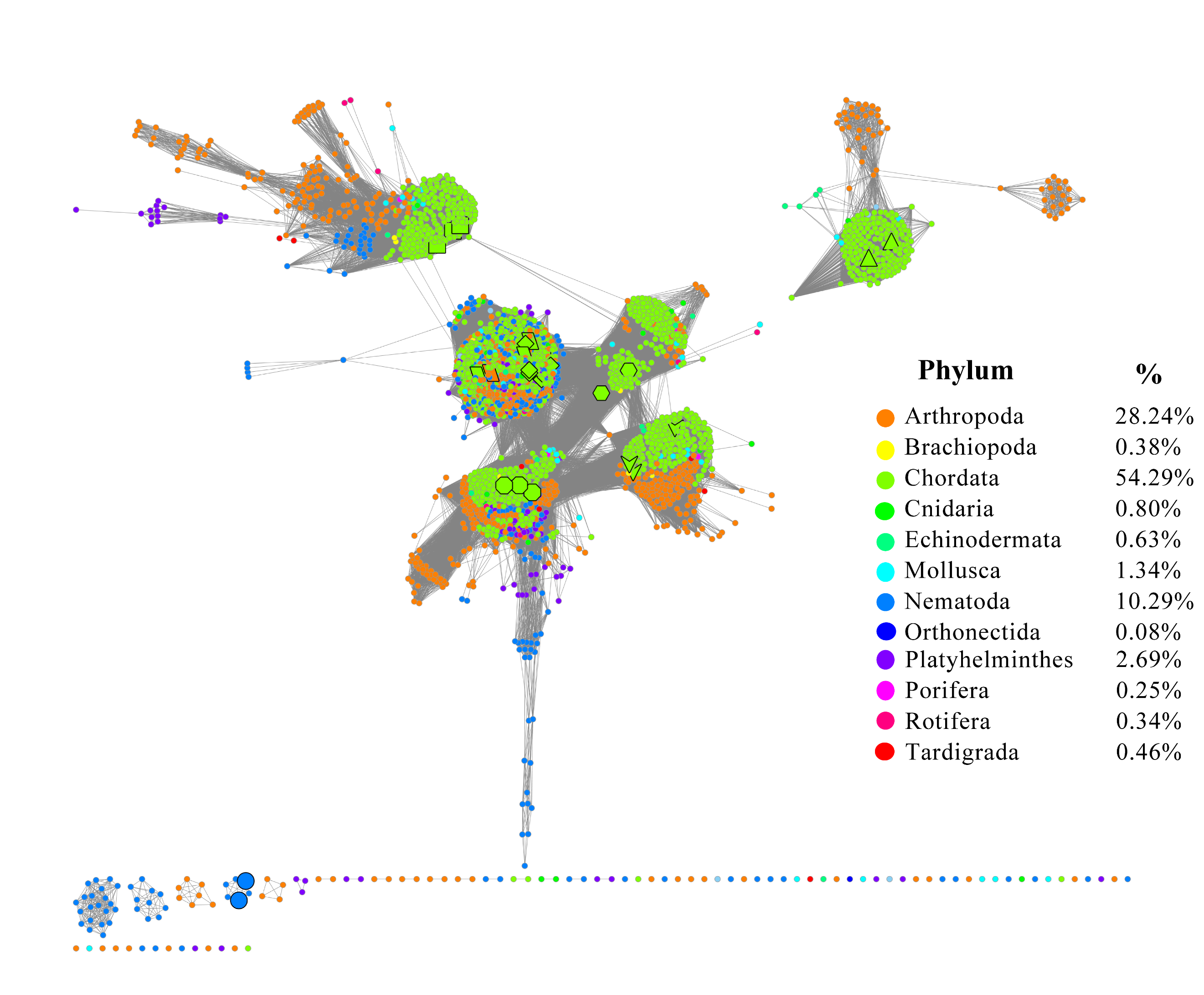


**Figure S1C**


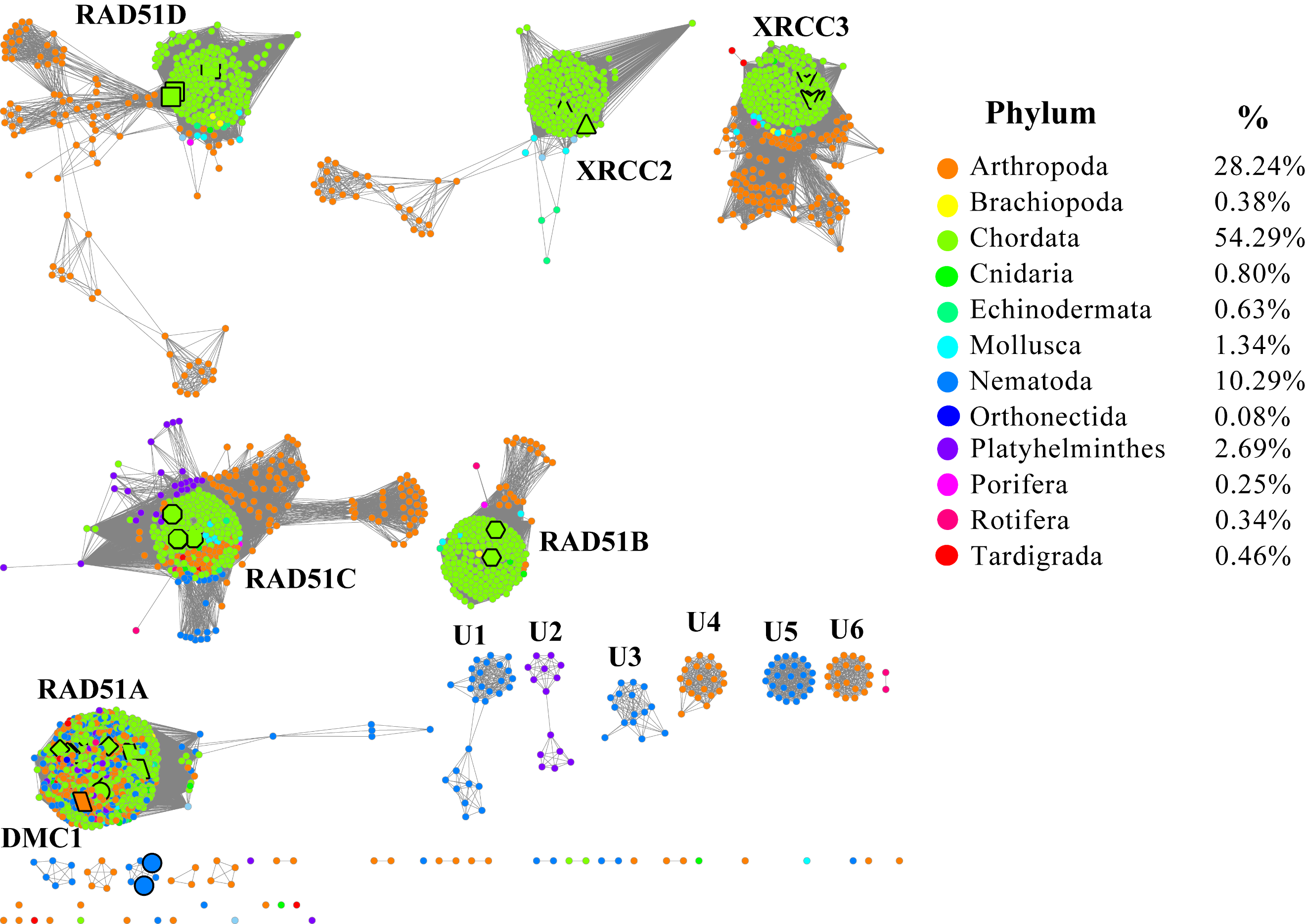


**Figure S1. The protein sequence similarity network (SSN) and taxonomic distribution (phylum) of RAD51 from Metazoa.** The sequence similarity network (SSN) of proteins in the InterPro database (IPR013632) was generated with an e-value threshold of 10^−20^ (A), 10^−20^ (B), and 10^−20^(C). Each node represents one protein. Edges are shown with BLASTP e-values below the indicated cutoff. A cluster was labeled if there were more than 10 nodes in it. RAD51A (diamond), RAD51B (hexagon), RAD51C (octagon), RAD51D (square), XRCC2 (triangle), XRCC3 (V), and DMC1 (parallelogram) are enlarged. The proteins that are annotated other names are also enlarged and shown in cycle forms. Nodes from the same taxonomic groups in the global network have the same color. The colors corresponding to each class and protein percentage in each class are listed at the right part.

**Figure S2**


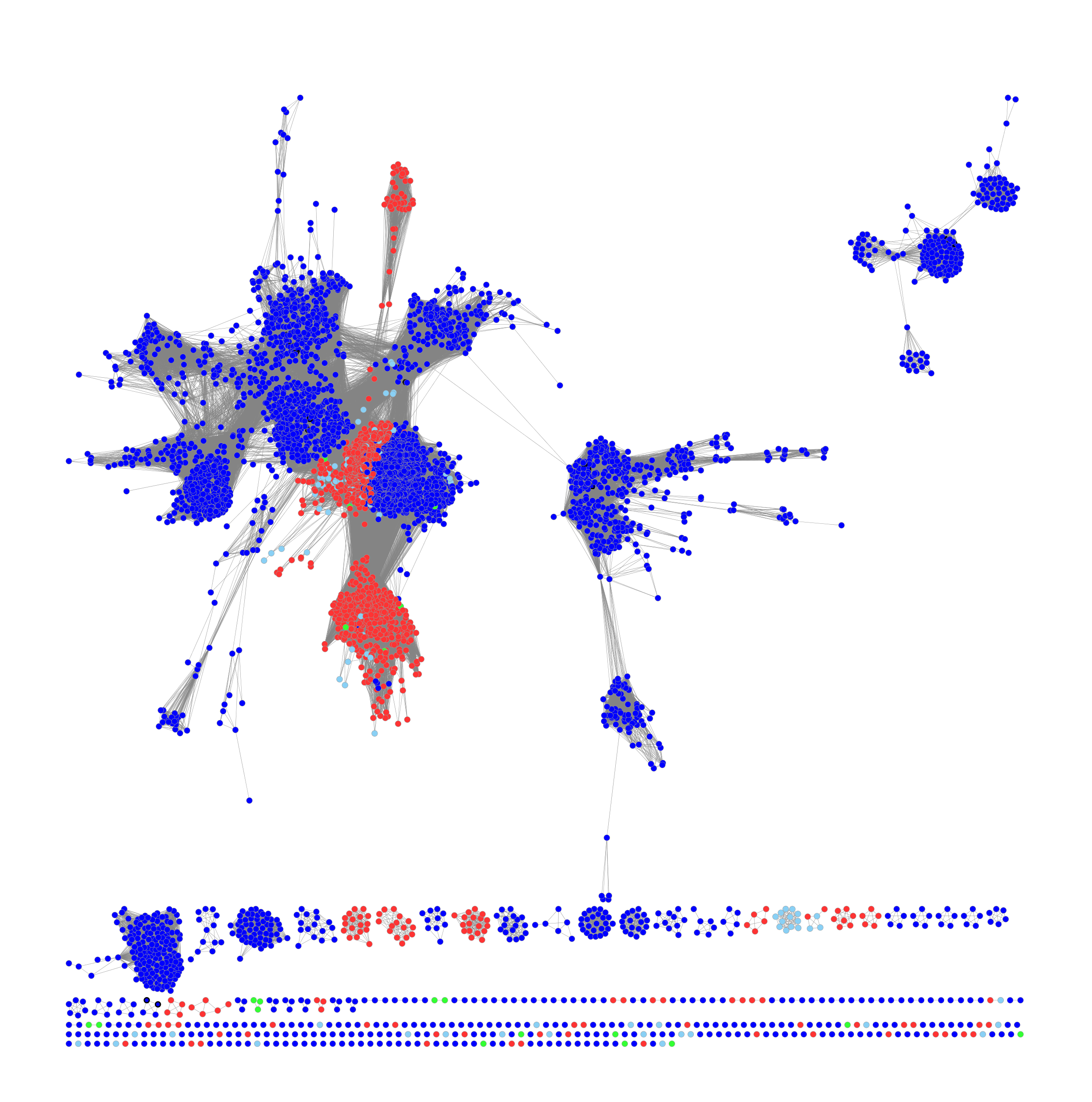


**Figure S2. The protein sequence similarity network (SSN) of RAD51 from** **eukaryotes and prokaryotes.** The sequence similarity network (SSN) of proteins was generated with an e-value threshold of 10^−25^. Each node represents one protein. Edges are shown with BLASTP e-values below the indicated cutoff. RAD51A (diamond), RAD51B (hexagon), RAD51C (octagon), RAD51D (square), XRCC2 (triangle), XRCC3 (V), and DMC1 (parallelogram) from human are enlarged and showed in yellow colour. Other proteins from Metazoa are shown in blue and the proteins in archaea are shown in red. The proteins from bacteria and metagenomes are shown in green and blue, respectively.
